# Prevalence of Coronary Heart Disease or Stroke Among Workers Aged <55 Years — United States, 2008–2012

**Published:** 2014-08-01

**Authors:** Sara E. Luckhaupt, Geoffrey M. Calvert

**Affiliations:** 1 Division of Surveillance, Hazard Evaluations, and Field Studies, National Institute for Occupational Safety and Health, CDC

Cardiovascular disease accounts for one in three deaths in the United States each year, and coronary heart disease and stroke account for most of those deaths (1). To try to prevent 1 million heart attacks and strokes by 2017, the U.S. Department of Health and Human Services launched the Million Hearts initiative, promoting proven and effective interventions in communities and clinical settings. In workplace settings, cardiovascular disease can be addressed through a Total Worker Health program, which integrates occupational safety and health protection with health promotion. To identify workers likely to benefit from such a program, CDC analyzed data from the National Health Interview Survey (NHIS) for the period 2008–2012 to estimate the prevalence of a history of coronary heart disease or stroke (CHD/stroke) among adults aged <55 years by selected characteristics, employment status, occupation category, and industry of employment. The results of that analysis showed that 1.9% of employed adults aged <55 years reported a history of CHD/stroke, compared with 2.5% of unemployed adults looking for work, and 6.3% of adults not in the labor force (e.g., unemployed adults who stopped looking for work, homemakers, students, retired persons, and disabled persons). Workers employed in service and blue collar occupations were more likely than those in white collar occupations to report a history of CHD/stroke. Two industry groups also had significantly higher adjusted prevalence ratios (aPRs) for CHD/stroke: Administrative and Support and Waste Management and Remediation Services* and *Accommodation and Food Service*.† Workers in these occupation and industry groups might especially benefit from a Total Worker Health approach to reducing the risk for CHD/stroke.

NHIS collects information about the health of the noninstitutionalized, civilian population in the United States, using nationally representative samples. Interviews are initiated in person with telephone follow-up when the interview cannot be completed in person. Questions about a history of CHD/stroke (defined as self-reported history of stroke or coronary heart disease [including angina and myocardial infarction] or both),§ employment status, industry, and occupation are asked of randomly selected adults. NHIS obtains verbatim responses from employed adult respondents (aged ≥18 years) regarding their industry (employer’s type of business) and occupation (employee’s type of work). These responses are reviewed by U.S. Census Bureau coding specialists, who assign 4-digit industry codes based on the 2007 North American Industrial Classification System.¶

For this analysis, an employment status variable was created with three categories: currently employed, unemployed, and not in the labor force. Adults were classified as unemployed if they reported that they were looking for work, whereas adults not in the labor force included unemployed adults who stopped looking for work, homemakers, students, retired persons, and disabled persons. Occupations were grouped into four categories: white collar, service (e.g., hairdresser, nurse’s aide, and cook), blue collar (e.g., construction worker, factory worker, and truck driver), and farm. For analyses by industry, the 21 simple 2-digit industry recodes provided in the NHIS public dataset were used, which are based on codes for the 2-digit North American Industrial Classification System. Industry categories with fewer than 10 cases were grouped into the *Other/Unknown* category.

Weighted data were used to produce national estimates. Point estimates and estimates of corresponding variances were calculated using statistical software to account for the complex sample design. Statistical significance was determined at p<0.05. Prevalence estimates were based on data collected by NHIS surveys during 2008–2012 from 91,331 adult respondents aged <55 years. Annual adult response rates ranged from 60.8% to 66.3%. For calculation of aPRs for industries, each category was compared with all other industry categories combined (e.g., workers employed in *Wholesale Trade Industries* were compared with workers employed in all other categories combined). To account for differences in workforce demographics by occupation and industry, aPRs based on occupation and industry were adjusted for sex and age. Differences in prevalence were considered significant if their 95% confidence intervals (CIs) did not overlap, and aPRs were considered significant if their CIs did not include 1.00.

The prevalence of a history of CHD/stroke among all adults aged <55 years was estimated to be 2.8% (Table 1), including 2.0% for coronary heart disease and 1.0% for stroke. The prevalence among employed adults was 1.9%. The prevalence was higher among both unemployed adults (2.5%) and adults not in the labor force (6.3%). Among adults who were employed, men and current and former smokers were significantly more likely than women and those who had never smoked to report a history of CHD/stroke. The prevalence of CHD/stroke among workers increased in each higher age group, and workers with a college degree were less likely than workers with less education to report a history of CHD/stroke.

After adjusting for sex and age, workers in service (aPR = 1.53) or blue collar (aPR = 1.40) occupations were more likely than those in white collar occupations to report a history of CHD/stroke (Table 2). Two industries had significantly higher adjusted aPRs: *Administrative and Support and Waste Management and Remediation Services* (aPR = 1.47) and *Accommodation and Food Service* (aPR = 1.37) (Table 3).

## Discussion

The aim of this study was to identify workers with a greater potential to benefit from programs designed to reduce the risk for CHD/stroke. Because age, the strongest predictor of CHD/stroke, cannot be modified, the study focused on prevalence differences among workers aged <55 years by selected characteristics, employment status, occupation category, and industry of employment. Although employed adults aged <55 years had a lower prevalence of a history of CHD/stroke than unemployed adults or adults not in the labor force, several groups of workers with higher prevalence of CHD/stroke were identified. The higher prevalence might, in part, be caused by occupational risk factors (i.e., characteristics of the workplace or job), but the prevalence of preexisting illness or risk factors among persons in different industries and occupations is unknown. Occupational CHD/stroke risk factors can include work stress (2), shift work (2), exposure to particulate matter (3), noise (4), and secondhand smoke (5). Health professionals and employers should take these factors into account when planning workplace interventions to prevent CHD/stroke. These factors might have both direct physiologic effects on cardiovascular health and indirect effects by influencing behavioral risk factors such as smoking and obesity. Some evidence indicates that workplace hazards such as job strain might pose more potent risk to workers in lower-income households, perhaps because of an interaction with adverse exposures in the community, combined with fewer health-enhancing opportunities (e.g., health care, a healthy diet, and exercise facilities) (6).

The industry and occupation categories found to be associated with a higher prevalence of CHD/stroke after adjustment for age and sex, *Administrative and Support and Waste Management and Remediation Services* and *Accommodation and Food Service* and service and blue collar occupations, are each characterized by multiple known CHD/stroke risk factors. For example, workers employed in *Administrative and Support and Waste Management and Remediation Services* industries have reported significantly higher rates of job insecurity, a common cause of job stress, compared with all workers combined (7). Workers employed in *Accommodation and Food Service* industries have been reported among those who are significantly more likely to work alternative shifts (8) and significantly more likely to smoke (9). Conversely, workers in industry groups with lower prevalence of CHD/stroke compared with all other workers (e.g., *Education Services* and *Information*) might be more likely to have access to preventive services and less likely to be exposed to occupational CHD/stroke risk factors.

The findings in this report are subject to at least four limitations. First, all results are based upon self-report of a history of CHD/stroke, which was not validated with medical records. Second, the broad industry and occupation categories used for this analysis aggregate workers who likely have substantially different working conditions. Third, this is a cross-sectional study; therefore, whether employment in any specific industry or occupation increases or decreases the risk for CHD/stroke cannot be determined. Finally, because the annual response rate was only 60.8%–66.3%, nonresponse bias might have affected the results.

Addressing the risk for CHD/stroke among workers might involve a Total Worker Health approach. Traditionally, health protection programs have focused squarely on safety, with the goal to reduce worker exposures to risk factors in the work environment, whereas workplace health promotion programs have focused exclusively on personal lifestyle factors. A growing body of science supports the effectiveness of combining these efforts through workplace interventions that integrate health protection with health promotion (10). CDC has developed several resources that might help employers implement Total Worker Health programs in their worksites.** This information is also important for clinicians, who should consider the potentially increased occupational risk for CHD/stroke in patients in certain industries and occupations and take their patients’ work status, workplace, and occupation type into account when developing prevention and treatment plans.

What is already known on this topic?

Cardiovascular disease (CVD) accounts for one in three deaths in the United States each year. In the workplace setting, CVD can be addressed through Total Worker Health, a program integrating occupational safety and health protection with health promotion to prevent worker injury and illness and to advance health and well-being. Coronary heart disease or stroke (CHD/stroke) account for the majority of deaths from cardiovascular disease.

What is added by this report?

Overall, 1.9% of employed adults aged <55 years reported a history of CHD/stroke, compared with 2.5% of unemployed adults looking for work, and 6.3% of adults not in the labor force. After adjusting for sex and age, workers in service and blue collar occupations, and those employed in two industries, *Administrative and Support and Waste Management and Remediation Services* and *Accommodation and Food Service*, had significantly higher prevalence of CHD/stroke.

What are the implications for public health practice?

Workers in the industry and occupation categories with increased risk for CHD/stroke might especially benefit from a Total Worker Health approach. Such an approach could integrate control of known occupational CHD/stroke risk factors with health promotion activities. Clinicians should consider the potentially increased risk for CHD/stroke in their working-age patients and take occupation and industry into account when developing prevention and treatment plans.

## Figures and Tables

**TABLE 1:** Prevalence of a history of coronary heart disease or stroke among adults aged <55 years, by selected characteristics and employment status — National Health Interview Survey, United States, 2008–2012

		All adults aged <55 yrs		Employed		Unemployed		Not in labor force	
					
Characteristic	No.	%	(95% CI)	%	(95% CI)	%	(95% CI)	%	(95% CI)
**Total respondents (2008–2012)**	**91,331**	**2.8**	**(2.7–3.0)**	**1.9**	**(1.8–2.1)**	**2.5**	**(2.2–3.0)**	**6.3**	**(5.8–6.8)**
**Survey year**									
2008	13,991	2.7	(2.4–3.1)	1.9	(1.6–2.3)	2.6	(1.4–4.7)	6.1	(5.1–7.3)
2009	17,928	2.9	(2.6–3.3)	2.1	(1.8–2.5)	2.5	(1.7–3.8)	6.1	(5.1–7.2)
2010	17,435	2.9	(2.6–3.3)	2.0	(1.7–2.3)	2.9	(2.1–4.0)	6.5	(5.5–7.7)
2011	20,793	2.7	(2.5–3.0)	1.8	(1.6–2.1)	2.5	(1.9–3.4)	5.9	(5.2–6.8)
2012	21,245	2.8	(2.5–3.1)	1.8	(1.6–2.1)	2.2	(1.5–3.0)	6.7	(5.7–7.9)
**Sex**									
Men	41,522	3.1	(2.9–3.4)	2.2	(2.0–2.5)	2.8	(2.2–3.5)	8.8	(7.8–9.9)
Women	49,870	2.5	(2.3–2.7)	1.6	(1.4–1.8)	2.3	(1.8–2.9)	5.0	(4.5–5.5)
**Age group (yrs)**									
<35	40,542	0.8	(0.7–1.0)	0.6	(0.5–0.8)	1.3	(1.0–1.8)	1.3	(1.1–1.7)
35–39	12,627	2.0	(1.7–2.3)	1.4	(1.2–1.7)	3.9	(2.5–6.1)	4.3	(3.4–5.4)
40–44	12,531	3.5	(3.1–3.9)	2.3	(1.9–2.8)	4.5	(3.1–6.5)	8.9	(7.2–10.9)
45–49	12,852	4.9	(4.5–5.4)	3.3	(2.9–3.8)	4.2	(2.9–6.1)	12.8	(11.2–14.6)
50–54	12,840	7.0	(6.4–7.6)	4.5	(4.0–5.1)	4.3	(2.9–6.4)	17.2	(15.4–19.1)
**Race/Ethnicity**									
White, non-Hispanic	48,673	2.8	(2.6–3.0)	2.0	(1.8–2.2)	3.0	(2.3–3.8)	6.3	(5.7–7.0)
Black, non-Hispanic	14,447	3.9	(3.5–4.3)	2.4	(2.1–2.9)	2.2	(1.6–3.0)	9.8	(8.6–11.1)
Asian, non-Hispanic	6,119	1.3	(1.0–1.7)	1.1	(0.8–1.5)	1.0	(0.4–2.5)	1.9	(1.1–3.2)
Other, non-Hispanic	2,124	4.2	(3.1–5.7)	2.4	(1.5–3.8)	1.7	(0.5–5.5)	10.2	(7.2–14.4)
Hispanic	20,029	2.3	(2.1–2.5)	1.6	(1.4–1.9)	2.3	(1.7–3.1)	4.3	(3.7–5.1)
**Education (age ≥25 yrs)**									
Less than high school diploma	11,042	5.4	(4.9–6.0)	2.7	(2.2–3.3)	4.5	(3.3–6.3)	10.9	(9.6–12.5)
High school diploma or GED certificate	18,705	4.5	(4.1–4.8)	2.9	(2.5–3.3)	3.9	(2.9–5.2)	10.7	(9.4–12.1)
Some college	23,184	3.5	(3.2–3.9)	2.5	(2.2–2.9)	3.0	(2.2–4.2)	8.6	(7.5–9.7)
College degree	23,672	1.4	(1.3–1.6)	1.4	(1.2–1.6)	1.6	(0.9–2.7)	2.2	(1.6–3.0)
**Household income (% of poverty level)**									
<100%	17,202	4.9	(4.4–5.4)	2.5	(2.0–3.0)	3.1	(2.5–3.9)	8.6	(7.6–9.7)
100%–199%	18,315	3.3	(3.0–3.7)	2.0	(1.7–2.3)	2.9	(2.0–4.1)	6.9	(6.0–8.0)
200%–399%	25,964	2.6	(2.4–2.9)	2.1	(1.8–2.3)	2.2	(1.5–3.2)	5.7	(4.7–6.8)
≥400%	28,933	1.9	(1.7–2.1)	1.7	(1.5–2.0)	1.5	(0.9–2.5)	3.0	(2.4–3.9)
**Health insurance**									
Yes	68745	2.9	(2.7–3.1)	1.9	(1.8–2.1)	2.4	(1.9–3.0)	7.0	(6.4–7.6)
No	22322	2.5	(2.3–2.8)	2.0	(1.8–2.3)	2.8	(2.2–3.4)	4.1	(3.4–4.8)
**Smoking status**									
Current smoker	20,366	4.4	(4.0–4.8)	2.8	(2.4–3.2)	3.0	(2.3–3.9)	10.6	(9.5–11.7)
Former smoker	13,219	4.6	(4.2–5.1)	3.2	(2.8–3.7)	4.2	(2.8–6.4)	12.0	(10.3–13.8)
Never smoker	57,305	1.8	(1.6–1.9)	1.3	(1.2–1.5)	1.9	(1.5–2.5)	3.4	(3.0–3.9)
**Length of unemployment** *									
<12 months	4,590	—	—	—	—	2.4	(2.0–3.0)	—	—
≥12 months	2,770	—	—	—	—	2.7	(2.1–3.6)	—	—

**Abbreviations:** CI = confidence interval; GED = General Educational Development.

* Counts only include unemployed adults.

**TABLE 2:** Prevalence of a history of coronary heart disease or stroke among adult workers aged <55 years, by occupation category — National Health Interview Survey, United States, 2008–2012

Occupation category	Unweighted sample size	Estimated no. of cases	% (95% CI)	aPR (95% CI)
White collar	37,416	1,057,000	1.6 (1.5–1.8)	Referent
Service	13,792	476,000	2.2 (1.8–2.6)	1.53 (1.27–1.85)
Farm	540	16,000	1.9 (1.0–3.6)	1.23 (0.63–2.41)
Blue collar	13,306	625,000	2.6 (2.3–3.0)	1.40 (1.19–1.65)

**Abbreviations:** aPR = adjusted prevalence ratio; CI = confidence interval.

**TABLE 3:** Prevalence of a history of coronary heart disease or stroke among employed adults aged <55 years, by industry category — National Health Interview Survey, United States, 2008–2012

Industry category*	Unweighted sample size	Estimated cases (in thousands)	Prevalence (95% CI)	aPR† (95% CI)
Wholesale Trade	1,560	81	2.9 (1.9–4.3)	1.34 (0.90–2.00)
Public Administration	3,356	152	2.8 (2.2–3.6)	1.22 (0.93–1.60)
Administrative and Support and Waste Management and Remediation Services	3,113	135	2.7 (2.1–3.5)	1.47 (1.11–1.96)
Transportation and Warehousing	2,617	123	2.7 (2.0–3.5)	1.14 (0.86–1.51)
Utilities	564	26	2.6 (1.4–4.8)	1.00 (0.54–1.86)
Real Estate and Rental and Leasing	1,188	51	2.5 (1.7–3.8)	1.17 (0.78–1.75)
Manufacturing	6,328	271	2.4 (1.9–3.0)	1.02 (0.82–1.27)
Agriculture, Forestry, Fishing, and Hunting	899	34	2.3 (1.4–3.6)	1.12 (0.70–1.77)
Other Services (except Public Administration)	3,269	118	2.2 (1.6–3.0)	1.20 (0.88–1.64)
Construction	4,261	169	2.1 (1.6–2.8)	0.97 (0.74–1.27)
Health Care and Social Assistance	9,381	286	1.9 (1.6–2.2)	1.12 (0.93–1.35)
Retail Trade	6,769	221	1.8 (1.5–2.2)	1.11 (0.90–1.37)
Accommodation and Food Service	4,978	147	1.8 (1.3–2.4)	1.37 (1.03–1.81)
Arts, Entertainment, and Recreation	1,332	41	1.8 (1.1–2.8)	0.99 (0.62–1.58)
Professional, Scientific, and Technical Service	4,417	115	1.5 (1.1–2.0)	0.71 (0.53–0.96)
Other/Unknown	1,701	37	1.4 (0.8–2.2)	0.64 (0.39–1.07)
Education Services	6,053	121	1.2 (0.9–1.5)	0.60 (0.46–0.78)
Information	1,564	30	1.1 (0.6–1.9)	0.58 (0.33–0.99)
Finance and Insurance	3,053	44	0.8 (0.5–1.3)	0.43 (0.27–0.70)

**Abbreviations:** aPR = adjusted prevalence ratio; CI = confidence interval.

* North American Industry Classification system, available at http://www.census.gov/cgi-bin/sssd/naics/naicsrch?chart=2007.

† For calculation of aPRs, each industry category was compared with all other industry categories.

## Footnotes

* Includes industries such as business support services, travel arrangements and reservation services, investigation and security services, services to buildings and dwellings (except cleaning during and immediately after construction), landscaping services, waste management, and remediation.

† Includes industries such as traveler accommodation, recreational vehicle parks and camps, rooming and boarding houses, restaurants and other food services, and bars.

§ Respondents were asked, “Have you ever been told by a doctor or other health professional that you had [condition]?” This question was asked separately for CHD, angina, myocardial infarction (heart attack), and stroke.

¶ Available at http://www.census.gov/cgi-bin/sssd/naics/naicsrch?chart=2007.

** Additional information available at http://www.cdc.gov/niosh/twh.
